# BCL-2 Expression in Primary Cutaneous Follicle Center B-Cell Lymphoma and Its Prognostic Role

**DOI:** 10.3389/fonc.2020.00662

**Published:** 2020-04-28

**Authors:** Alessandro Pileri, Claudio Agostinelli, Clara Bertuzzi, Vieri Grandi, Vincenza Maio, Irene Lastrucci, Marco Santucci, Nicola Pimpinelli

**Affiliations:** ^1^Dermatology Unit, Department of Experimental, Diagnostic and Specialty Medicine, University of Bologna, Bologna, Italy; ^2^Hematopathology Unit, Department of Experimental, Diagnostic and Specialty Medicine, University of Bologna, Bologna, Italy; ^3^Dermatology Unit, Department of Health Sciences, University of Florence Medical School, Florence, Italy; ^4^St John's Institute of Dermatology, Guy's and St Thomas' Hospitals NHS Foundation Trust, London, United Kingdom; ^5^Pathological Anatomy Unit, Department of Health Sciences, University of Florence Medical School, Florence, Italy

**Keywords:** cutaneous B-cell lymphoma, follicle center B-cell lymphoma, BCL-2, prognosis, immunohistochemistry

Primary cutaneous lymphomas (PCLs) are a heterogeneous group of lymphoid neoplasms showing peculiar clinical, histologic, immunohistochemical, and molecular features. PCLs encompass two groups: primary cutaneous B-cell lymphomas (PCBCLs) and primary cutaneous T-cell lymphomas (PCTCLs). Globally, PCLs represent the 19% of all extra-nodal non-Hodgkin's lymphomas. An American population-based study on PCLs diagnosed between 2001 and 2005 has revealed a dramatic increase in the prevalence of PCLs over the past three decades, varying from 5.0/1 million person-years in the early 1980s to 12.7 in 2004–2005 (1). The incidence of PCTCL is higher than that of PCBCL [incidence ratio (IR): 7.7 vs. 3.1/1 million person-years, respectively], while male gender is the most affected (14.0 vs. 8.2/1 million person-years). The IR of PCTCL is highest among black people (10.0/1 million person-years), whereas non-Hispanic whites are most affected by PCBCL (IR: 3.5/1 million person-years).

PCBCLs have been categorized in the 2005 WHO-EORTC classification into three types: primary cutaneous marginal zone lymphoma (PC-MZL), primary cutaneous follicle center lymphoma (PC-FCL) and primary cutaneous diffuse large B-cell lymphoma (PC-DLBCL), leg type (2). Such a classification is still accepted albeit some changes (i.e., primary cutaneous marginal zone lymphoma included within the group of extranodal marginal zone lymphomas of mucosa associated lymphoid tissue) have occurred during the past 15 years or new provisional entities have been proposed (i.e., EBV+ mucocutaneous ulcer) (3, 4). Despite the difference between PC-MZL, PC-FCL and PC-DLBCL have been exhaustively investigated, an animate debate on BCL-2 expression in PC-FCL is present in the literature. BCL-2 is an antiapoptotic molecule and exerts its function by blocking pro-apoptotic stimuli leading to neoplastic cells survival, gaining advantages to tumor growth and spread (5). BCL-2 molecule is expressed in about 90% of systemic FCLs that correspond to a separate entity from primary cutaneous cases. Due to the possibility of a skin-involvement by a systemic form (on a clinical ground it is impossible to distinguish the two forms), the differentiation of a primary cutaneous from a nodal disease is not trivial and have an impact on a prognostic a therapeutic grounds (i.e., better/worse clinical outcome and skin-directed/systemic treatment, respectively). Furthermore, BCL-2 is strongly expressed by PC-DLBCL, leg type which is characterized by a more aggressive clinical course. However, the distinction between a PC-FCL and a PC-DLBLC can be based also on a different clinical presentation.

## Primary Cutaneous Follicle Center Lymphoma

PC-FCL, the most common PCBCL, encompasses approximately 50–60% of all PCBCL cases. PC-FCL can occur in both genders mostly in their 50s (3, 6, 7). The disease clinically is characterized by erythematous to violaceous plaque and tumor, with a single or multiple-lesion presentation. The most common affected areas are the head & neck and trunk. The tumors on the trunk can be surrounded by erythematous patches or plaques, which can precede the development of tumors by months or years. At histology, PC-FCL shows an infiltrate of neoplastic cells with a follicular, follicular and diffuse, or diffuse growth pattern. Cases with a follicular growth pattern can present with involvement of the entire dermis extending to the subcutis. Reactive T-cells and a prominent stromal component are usually detected. Cases with a diffuse growth pattern are characterized by the presence of a monotonous population of large centrocytes admixed with a variable number of centroblast (3, 6, 8). The neoplastic infiltrate usually spares the epidermis (3, 6). On immunohistochemistry, B-cell markers such as CD20, PAX5, and BCL-6 turn out to be positive (9, 10), while staining for CD5 and CD43 molecule is negative. Most cases show an IRF4 and FOXP1 negativity, while CD10 and BCL-2 expression is usually absent. However, several studies (see section below) have reported a variable BCL-2 expression in PC-FLC cases. Most cases presented with at least partially follicular growth pattern (7, 9, 11–20). However, BCL-2 and CD10 strong expression should alert clinician of the possibility of a skin involvement by a nodal disease and an adequate staging is required. PC-FCL has an excellent prognosis with a 5-year overall survival over 95%. The disease can be successfully treated with surgical removal of the lesions or limited-field radiotherapy treatment (3, 6, 10).

## BCL-2 Expression in Primary Cutaneous Follicle Center Lymphoma

Pioneer studies on PC-FCL have highlighted that the absence of BCL-2, CD10, MYC, and IRF4 expression on immunohistochemistry as well as the lack of t (14, 18) at polymerase chain reaction (PCR) or fluorescence *in situ* hybridization (FISH) should be regarded as helpful tools for the differential diagnosis between indolent PC-FCL and aggressive diseases (systemic FCL with skin involvement; PC-DLBCL, leg type) (2, 9). The differential diagnosis between indolent and aggressive PCBCL variants or cutaneous and systemic diseases is crucial to administer the appropriate treatment.

PC-FCL cases with BCL-2 expression on immunohistochemistry or translocations of *BCL2* at FISH have been described (7, 11, 19). Those cases showed the usual indolent behavior of “conventional” PC-FCL cases, an accurate staging (i.e., whole body CT/PET scan and bone marrow biopsy) and follow up being the most important recommendation (7, 9, 11–20).

Different authors found BCL-2 positivity as well as t (14, 18) at PCR in a variable proportion of their cases (16). The same Groups observed BCL-2 expression and t (14, 18) mostly in low-grade PC-FCL, suggesting that those abnormalities may not be related with an aggressive behavior. Two Groups have speculated whether PC-FLC and systemic FCL with a skin involvement may have a quite close pathogenesis, owing to the expression of BCL-2 on immunohistochemistry and the *BCL-2* translocation at PCR, both in cutaneous and systemic FCL featuring a skin colonization (11, 13, 15). However, a long-term follow up was not provided making impossible to draw any conclusion on any difference on a long-term clinical behavior between BCL-2+/BCL-2- cases as well as those with or without t (14, 18) translocation. Another variable has been introduced by the use of the FISH technique instead of PCR, the latter being less sensible in detecting t (14, 18) translocation (17). Recently, two Groups have validated literature data on BCL-2 expression on immunohistochemistry and provided a possible explanation of the variable proportion of positive cases in the literature (BCL-2+ cases from 0 to 86%) (7, 18). The Spanish group (7) hypothesized that improvement of antigen retrieval techniques may be the rationale for the non-homogeneous literature findings on BCL-2 expression. Moreover, the heterogeneous data on t (14, 18) may be explained by the use of different techniques (FISH or PCR) (19, 21). Servitje et al. (7) concluded that genetic and immunohistochemical abnormalities should not be regarded as marker of a worse prognosis.

As stated before the differential diagnosis between a PC-FCL and a PC-DLBCL can be easier, owing to the different clinical presentation (plaques and tumors usually involving the leg of a female in her 70s) and the strong positivity of BCL-2 and IRF4/MUM1 molecules on immunohistochemistry. Moreover, PC-DLBCL can show double BCL-2 and MYC expression a finding not observed in PC-FLC.

## Conclusions

In conclusion, the detection of the BCL-2 molecule by immunohistochemistry in PC-FLC should not surprise clinicians. Furthermore, t (14, 18) translocation can be observed especially at FISH analysis, which can be more reliable and accurate in detecting gene abnormalities than PCR. Due to the absence of long-term follow up studies, clinicians should accurately stage and follow up patients, albeit there is no clear-cut evidence supporting a worse prognostic significance. Essentially, the accurate stage and follow up is meant to avoid the risk to misdiagnose a systemic form as a PC one. More and possibly perspective studies should be warranted to better define the clinical and biological landscape of PC-FCL.

## Author Contributions

AP, CA, CB, VG, VM, IL, MS, and NP: substantial contributions to the conception of the work, drafting the work and revising it critically for important intellectual content, and approval for publication of the content.

## Conflict of Interest

The authors declare that the research was conducted in the absence of any commercial or financial relationships that could be construed as a potential conflict of interest.
